# APOBEC3B up-regulation independently predicts ovarian cancer prognosis: a cohort study

**DOI:** 10.1186/s12935-018-0572-5

**Published:** 2018-05-29

**Authors:** Yan Du, Xiang Tao, Jing Wu, Huandi Yu, Yinhua Yu, Hongbo Zhao

**Affiliations:** 1 Obstetrics and Gynecology Hospital, Fudan University, 419 Fangxie Rd, 200011 Shanghai, People’s Republic of China; 2 The Academy of Integrative Medicine of Fudan University, 200011 Shanghai, People’s Republic of China; 3 Shanghai Key Laboratory of Female Reproductive Endocrine Related Diseases, 200011 Shanghai, People’s Republic of China

**Keywords:** APOBEC3B, Expression, Ovarian cancer, Survival, Viability

## Abstract

**Background:**

Ovarian cancer is a heterogeneous disease with a high degree of genomic instability, pro-/antitumor immunity and inflammation, and remains the most lethal gynecologic cancer worldwide. APOBEC3B, a member of the AID/APOBEC family, is part of the innate immune system which plays a key role in combating exogenous infection especially viral infection. Studies have shown that APOBEC3B expression is elevated in a variety of cancer tissues and cell lines, and plays a prominent role in the genesis and evolution of various cancers. However, the clinical relevance of APOBEC3B in ovarian cancer needs to be further investigated. The current study aimed to evaluate the predictive value of APOBEC3B in ovarian cancer clinical outcome, and to explore possible molecular mechanisms contributing to ovarian cancer progression.

**Methods:**

The expression of APOBEC3B in biopsy tissue specimens from 88 ovarian cancer patients was examined using immunohistochemistry. In addition, ovarian cancer cell lines were transfected with APOBEC3B siRNA or pLenti-APOBEC3B construct. Western blotting and SRB assay were performed to explore the role of APOBEC3B in ovarian cancer.

**Results:**

Patients were followed for a median of 74.77 months following the time of surgery. Forty-two patients had died, 5 had relapsed but were still alive at the end of study, and 41 patients remained alive and had no recurrence. Over-expression of APOBEC3B was associated with advanced FIGO stage and elevated CA125 (both p< 0.05). Univariate analysis result showed that histological subtype, FIGO stage, intravascular tumor thrombus, CA125 and *APOBEC3B* expression were associated with overall survival and disease-free survival of ovarian cancer patients. Multivariate analysis result showed that higher *APOBEC3B* expression were an independent prognostic factor to predict both worse overall survival (hazard ratio: 5.18, 95% confidence interval: 1.40–11.95, p= 0.003) and disease-free survival (hazard ratio: 4.23, 95% confidence interval: 1.60–11.17, p= 0.004) of ovarian cancer patients. Furthermore, knockdown of *APOBEC3B* expression in ovarian cancer cells caused an decrease in cell line viability.

**Conclusions:**

*APOBEC3B* expression is an independent prognostic factor in ovarian cancer patients. Knockdown of *APOBEC3B* expression affects ovarian cancer viability.

## Background

Ovarian cancer is one of the most common gynecologic cancer among women and the most lethal gynecologic malignancy worldwide [1]. There were estimated 238,700 new cases of and 151,900 deaths due to ovarian cancer in 2012 [1]. In China, ovarian cancer burden will stay stable due to the aging population [2]. The etiology of ovarian cancer remains unclear. Epidemiological studies have shown that several factors, including family history, personal history of breast cancer, menstrual and reproductive factors, obesity, hormone therapy [3], inflammation, as well as genetic mutations (*BRCA1* and *BRCA2* mutations) are associated with ovarian cancer risk [4]. In addition, survival rate of ovarian cancer patients has improved little over the last 30 years [5], with a reported 5-year survival of 45% in the United States [6]. The current mainstay treatment strategies for ovarian cancer are surgery followed by chemotherapy. Most ovarian cancers are diagnosed at the late stages with a poor prognosis due to advanced disease and chemotherapy resistance. There is a lack of reliable screening strategy for ovarian cancer. Therefore, it is important to investigate the molecular mechanisms and key regulators of ovarian cancer progression and metastasis in order to identify novel biomarkers to predict survival and recurrence risk, which will enable the selection of optimal treatment strategies and eventually improve prognosis.

In humans, the AID/APOBEC family has eleven members including activation-induced cytidine deaminase (AID, gene name: *AICDA*), and apolipoprotein B mRNA editing enzymes, catalytic polypeptide-like [APOBECs: APOBEC1 (A1), APOBEC2 (A2), APOBEC3 (A3), APOBEC3A (A3A), APOBEC3B (A3B), APOBEC3C (A3C), APOBEC3DE (A3DE), APOBEC3F (A3F), APOBEC3G (A3G), APOBEC3H (A3H), and APOBEC4 (A4)] [7]. It can specifically edit DNA or RNA through the irreversible cytidine and deoxycytidine deamination, causing the conversion of target cytosine (C) to uracil (U), and consequently causing DNA or RNA alterations/damages [8]. Studies have shown that APOBEC contribute to malignant transformation [9]. Two meta-analyses based studies independently identified a link between deleterious somatic mutations with cytosine mutation bias in different cancer types, as well as APOBEC expression/enzymatic activities [10, 11]. Of particular interest is APOBEC3B, one member of the APOBEC3 subfamily, which is responsible for the majority of cytosine mutations [12, 13]. It was also suggested that APOBEC3B may be a potential prognostic marker and therapeutic target for breast cancer [12]. A recent study proposed an AID/APOBEC-derived survival model for ovarian cancer risk assessment and revealed that the network is particularly associated with remodeling/fibrotic pathways, altered immune response, and autoimmune disorders with inflammatory background [14]. Another study showed that APOBEC3B overly expressed in the majority of ovarian cancer cell lines and high-grade primary ovarian cancers. In addition, it also showed that *APOBEC3B* expression positively correlated with total mutation load as well as elevated levels of transversion mutations [15]. However, the clinical relevance of APOBEC3B in ovarian cancer needs to be further investigated. The current study aimed to evaluate the predictive value of APOBEC3B in ovarian cancer prognosis, and to explore the oncogenic role of APOBEC3B in ovarian cancer.

## Materials and methods

### Study population

This retrospective cohort study was conducted at the Obstetrics and Gynecology Hospital of Fudan University in Shanghai, China. During May 2006 to November 2008, patients with newly diagnosed sporadic ovarian cancer were selected. The study protocol was approved by the institutional review board of the hospital (Reference Number: 2018-24). Written informed consent was obtained from all participants.

### Follow-up after surgical treatment

Clinical data and histopathologic data were collected from patients’ clinical and pathologic records. Follow-up was started 6 months after the surgery. Follow-up was performed by examinations every 3 months on an outpatient bases and/or by telephone calls according to our standard epidemiologic protocol.

### TMA construction and immunohistochemistry

Tissue microarrays (TMAs) containing the specimens were obtained from the Tissue Bank of Obstetrics and Gynecology Hospital, Fudan University. Formalin-fixed, paraffin blocks of 112 ovarian cancer patients were retrospectively collected. Each specimen has the information of age, laterality, histology, and Federation of Gynecologists and Obstetricians (FIGO) stage at surgery. Before construction of TMA, hematoxylin and eosin (HE) stained slides from the blocks were reviewed to circle the interested region for coring. A 1-mm-diameter core in the circling region was released with a needle from each block, arrayed, and re-embedded in a recipient block. For standardization between different recipient blocks, in-cohort controls of the cores from eight specimens were presented on every array-block. To ensure representativeness, there were 2–3 cores for each patient on the array-block. After TMA construction, a HE section of the recipient block was reviewed to confirm that the interested region was contained in the cores. TMA cutting was performed and all finished slides were dipped in paraffin for preservation at 4 °C before immunohistochemistry assays.

The expression of APOBEC3B in each pretreatment biopsy tissue specimen was examined using immunohistochemistry according to previous protocol [16]. Briefly, the de-paraffinized sections were incubated with 20% goat serum for 30 min to block non-specific binding and were then incubated with primary antibodies against APOBEC3B (Abacam, Cambridge, UK, 1:100) at 4 °C overnight followed by anti-rabbit secondary antibody for 1 h at 37 °C. Bound antibody was then visualized using the EnVision™ Detection Systems (Dako, Glostrup, Denmark). Scoring the intensity of immunostaining was done semiquantitatively, and immunostaining was scored based on the percentage of the stained tumor cells: 0–10% as negative (−), 11–25% as slightly positive (+), 26-50% as moderately positive (++), and 51-100% as strongly positive (+++), respectively [17]. Those scores were then categorized into lower expression (− and +) and higher expression (++ and +++) for analyses. All specimens were analyzed independently by two observers (YD and HZ) who were blinded to the clinical information. There was close agreement (> 90%) among the two investigators. Disagreements were resolved by consensus.

### Reagents and antibodies

Monoclonal antibodies to APOBEC3B and GAPDH were obtained from Abcam (Abcam, Cambridge, UK). Secondary antibodies conjugated with horseradish peroxidase (HRP) were purchased from Jackson ImmunoResearch Laboratories (Jackson ImmunoResearch Laboratories, West Grove, PA, USA). Human pLenti-APOBEC3B construct was obtained from Asia-Vector Biotechnology (Asia-Vector Biotechnology, Shanghai, China).

### Cell lines and culture

The four human ovarian cancer cell lines, A2780, HO-8910PM, SKOV3, and HEY were obtained from MD Anderson Cancer Center and the original source being the American Type Culture Collection (ATCC) (Manassas, VA, USA). All cell lines were cultured in 1640 complete medium supplemented with 10% FBS in 5% CO_2_ at 37 °C.

### Transfection of siRNA and pLenti-APOBEC3B construct

All cells were seeded in 60 mm dishes at a density of 2 × 10^5^/well, and transfected with APOBEC3B siRNA or pLenti-APOBEC3B construct using Lipo 2000 reagent according to the manufacturer’s instructions. The siRNAs targeting APOBEC3B and scrambled siRNA were synthesized and purified by RioBio Co. (RioBio Co., Guangzhou, China). At 48 h post-transfection, the cell pellets were collected for western blotting analysis. The target sequence of APOBEC3B siRNA 1 is 5′-GGUGUAUUUCAAGCCUCAG-3′, and the target sequence of APOBEC3B siRNA 2 is 5′-CCUGAUGGAUCCAGACACA-3′.

### Western blotting

All cells were lysed in 1×SDS lysis buffer (50 mM Tris–HCl, pH 6.8, 2% SDS, 10% glycerol, 1 mM PMSF, and 1 mM Na3VO4) and performed as described previously [18]. A total of 50 μg protein per lane was loaded on an SDS-PAGE gel and transferred to PVDF membrane (Millipore Corporation, USA). After being blocked with PBS containing 5% BSA and 0.05% Tween 20, the membrane was incubated with specific primary antibodies and followed by incubation with HRP-conjugated secondary antibodies (Jackson ImmunoResearch Laboratories, West Grove, PA). The labeled proteins were then visualized by fluorography using an enhanced chemiluminescence system (Thermo Scientific, Pierce Biotechnology, USA).

### Sulforhodamine B cell proliferation assay

The Sulforhodamine B (SRB) cell proliferation assay was conducted as previously described [18]. Briefly, 1 × 10^5^ of each four cells were plated in a 6-well plate and transfected with APOBEC3B siRNA or scrambled siRNA. At 24 h post-transfection, cells were seeded in a 96-well plate for the indicated time points and then fixed for 1 h at 4 °C by adding 50 μl of 30% (v/v) trichloroacetic acid. After fixation, the cells were washed five times using distilled water and stained with 100 μl of 0.4% (w/v) SRB in 1% (v/v) acetic acid solution for 30 min at room temperature. The cells were washed five times with 1% acetic acid solution and air-dried. The stain was solubilized in 100 μl of 10 mM Tris (pH 10.5), and the absorbance was measured at 570 nm using a microplate reader (Infinite M200, Tecan, Austria).

### Statistical analysis

For patient characteristics, mean and standard deviation (SD) were calculated for continuous variables, and number and percentage were calculated for categorical variables. Survival time was defined as the time from surgery until the patient’s death/recurrence, or the last time that the patient was known to be alive. Kaplan–Meier method was used to estimate the overall survival (OS) and disease-free survival (DFS). Log-rank test was used to compare the curves of different *APOBEC3B* expression groups. Cox proportional hazards models were used to estimate the survival distributions, hazard ratios (HRs) and corresponding 95% confidence intervals (95% CIs). Only significant prognostic variables in univariate analyses were included in the multivariate analyses. Forward Wald test was used in multivariate Cox regression models to determine independent prognostic factors. All significance tests were two sided; p value of < 0.05 was considered as statistically significant. Data analyses were performed by SPSS 16.0 for Windows (SPSS Inc., Chicago, IL, USA).

## Results

### Clinicopathological characteristics and survival of ovarian cancer patients

In total, 112 ovarian cancer patients who underwent surgery at our hospital were evaluated in this study. TMAs containing ovarian cancer specimens from 112 cases were used. Tissue samples from 24 cases were lost during cutting, leading to 88 ovarian samples for the present study. Table 1 shows the baseline clinicopathological characteristics of the study population. The average age of the patients was 51.42 ± 9.34, with the range of 26–74 years. More than half of the patients had serous ovarian carcinoma (n = 59, 67.0%). The majority of the patients had advanced-stage ovarian cancer (FIGO III and IV: n = 50, 56.8%) at initial diagnosis. After a median observation of 74.77 months (range 1.60–103.13 months), 42 patients had died, 5 had relapsed but were still alive at the end of study, and 41 patients remained alive and were free of ovarian cancer.

**Table 1:** Clinicopathological characteristics of study patients (n = 88)

Variables	N	%
Age at diagnosis (years) Median (range)	51	26–74
Age (years)		
≤ 50	41	46.6
> 50	47	53.4
Overall survival (months)		
Median (range)	74.77	1.60–103.13
Number of deaths	42	47.7
Disease free survival (months)		
Median (range)	67.65	0.80–103.13
Number of recurrences	46	52.3
Menopause		
Yes	45	51.1
No	43	48.9
Laterality		
Right side	23	26.1
Left side	16	18.2
Bilateral	49	55.7
Behavior		
Borderline	0	0
Invasive	88	100
Histological subtype (%)		
Serous	59	67.0
Non-serous	29	33.0
Histological grade (%)		
G1	8	9.1
G2	21	23.9
G3	37	42.0
Missing	22	25.0
FIGO (%)		
I	25	28.4
II	13	14.8
III	45	51.1
IV	5	5.7
Intravascular tumor thrombus (%)		
Yes	16	18.2
No	69	78.4
Missing	3	3.4
Serum CA 125 (U/mL)		
< 35	11	12.5
≥ 35	63	71.6
Missing	14	15.9
Serum CA 19-9 (U/mL)		
< 37	38	43.2
≥ 37	18	20.5
Missing	32	36.4
Serum CEA (ng/mL)		
< 5	58	65.9
≥ 5	2	2.3
Missing	28	31.8
Chemotherapy		
Yes	83	94.3
Missing	5	5.7
Traditional Chinese medicine treatment		
Yes	18	20.5
No	70	79.5
APOBEC3B		
−	8	9.1
+	32	36.4
++	32	36.4
+++	16	18.2

*APOBEC3B* apolipoprotein B mRNA editing enzyme catalytic subunit 3B, *CA 125* cancer antigen 125, *CA 19*-*9* cancer antigen 19-9, *CEA* carcinoembryonic antigen, *FIGO* Federation of Gynecologists and Obstetricians

### Association of *APOBEC3B* expression with clinicopathological factors

**Fig. 1:**
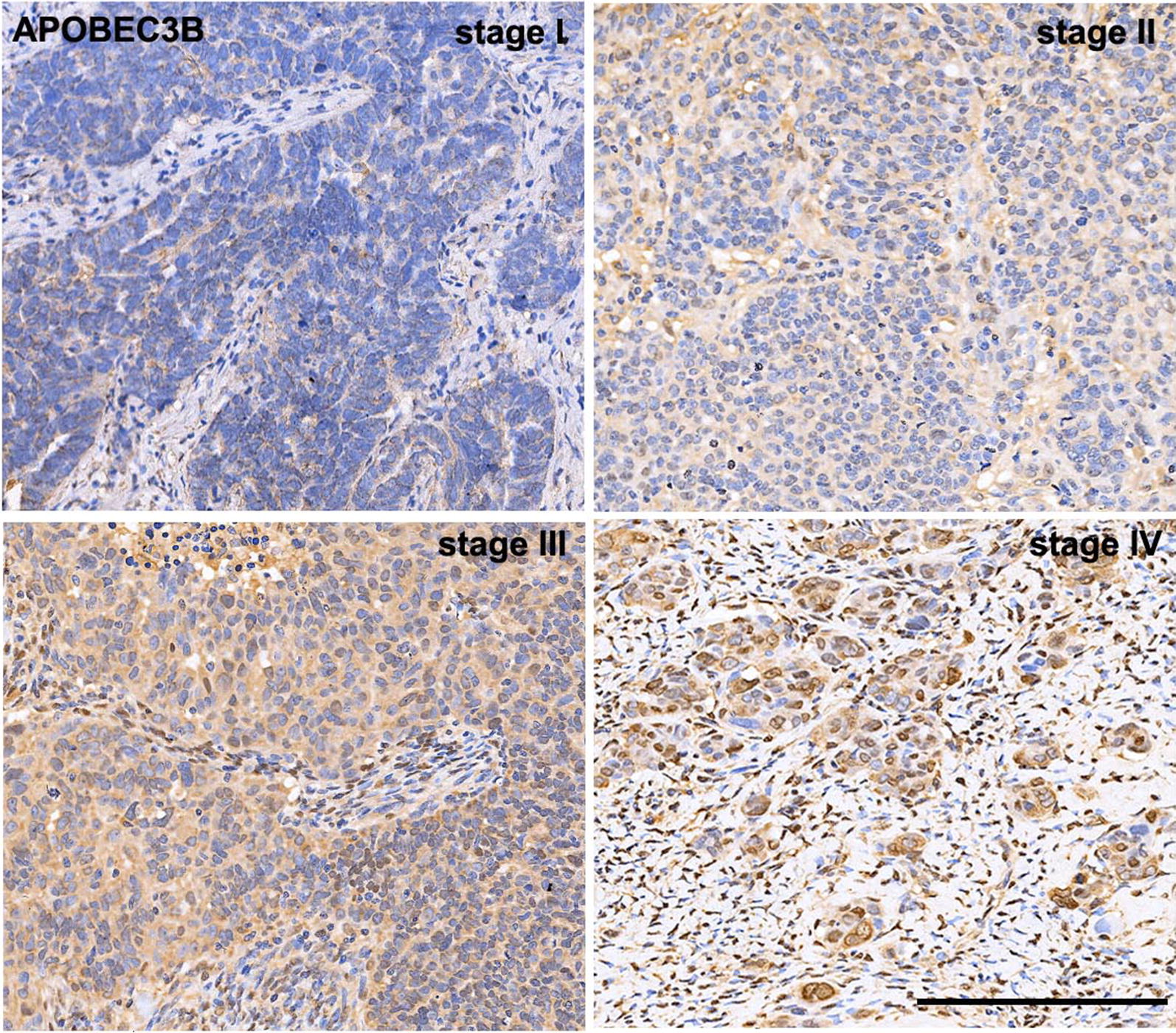
Representative immunohistochemical data of *APOBEC3B* expression in ovarian cancer TMA (FIGO stage I, II, III, and IV). Expression of APOBEC3B protein localizes in both nuclear and cytoplasmic compartment, and APOBEC3B has a higher ratio of cytoplasmic localization, as indicated by the brown color (scale bar, 200 μm)

Unlike a previous study which reported that APOBEC3B protein predominantly localized to the nuclear compartment in ovarian cancer cell lines [15], our immunostaining data from ovarian cancer tissues showed that APOBEC3B localized in both nuclear and cytoplasmic compartment (Fig. 1). Our immunohistochemistry results also showed that APOBEC3B has a higher ratio of cytoplasmic localization in ovarian cancer tissues (Fig. 1). The association between *APOBEC3B* expression and clinicopathological variables are presented in Table 2. Briefly, over-expression of APOBEC3B protein was significantly associated with advanced FIGO stage and elevated CA125 (both p< 0.05). There was no significant association between *APOBEC3B* expression and other clinicopathological factors.

**Table 2:** Association of *APOBEC3B* expression with clinicopathological parameters of ovarian cancer

Variables	APOBEC3B		p value
	∓	++/+++	
Age			0.558
≤ 50	20	21	
> 50	20	27	
Histological subtype			0.408
Serous	15	14	
Non-serous	25	34	
FIGO stage			*0.001*
I/II	25	13	
III/IV	15	35	
Histological grade*			0.976
G1	4	4	
G2	10	11	
G3	17	20	
Intravascular tumor thrombus^a^			0.455
No	33	36	
Yes	6	10	
CA 125^b^			*0.004*
< 35 U/mL	9	2	
≥ 35 U/mL	22	41	

*CA 125* cancer antigen 125, *FIGO* Federation of Gynecologists and Obstetricians

* Number of missing value = 22

^a^Number of missing value = 3

^b^Number of missing value = 14

### Association of *APOBEC3B* expression with survival of ovarian cancer patients

**Fig. 2:**
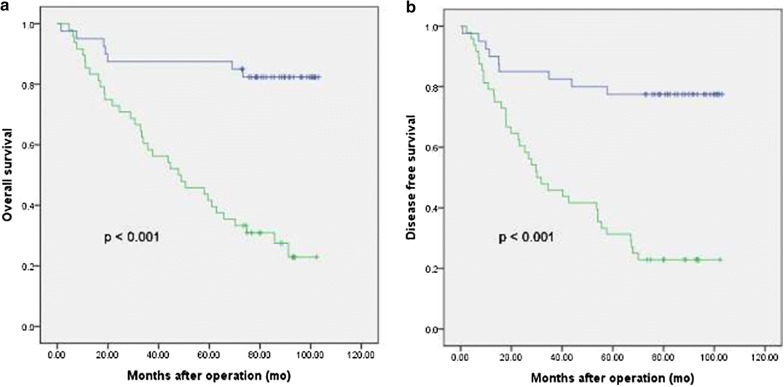
Kaplan-Meier survival curve. **a** Overall survival; **b** Disease-free survival stratified by APOBEC3B expression in 88 ovarian cancer patients

Patients with APOBEC3B-overexpressing tumors had both worse OS and DFS compared with those without or with lower APOBEC3B-expressing tumors (both p< 0.001; Fig. 2a, b). Univariate analyses showed that serous histological subtype (serous vs. non-serous: HR 2.24, 95% CI 1.12–4.47, p = 0.023), higher FIGO stage (III/IV vs. I/II: HR 7.89, 95% CI 3.53–17.60, p < 0.0001), intravascular tumor thrombus (Yes vs. No: HR 2.27, 95% CI 1.20–4.29, p= 0.012), elevated serum cancer antigen (CA) 125 (≥ 35 U/mL vs. < 35 U/mL: HR 5.45, 95% CI 1.32–22.56, p= 0.019) and over-expression of APOBEC3B (+++/++ vs. ± : HR 6.34, 95% CI 2.80–14.38, p< 0.0001) were significantly associated with a shorter OS (Table 3). Multivariate analysis revealed that higher FIGO stage (III/IV vs. I/II: HR 7.89, 95% CI 2.33–26.70, p= 0.001) and higher expression of APOBEC3B (+++/++ vs. ± : HR = 5.18, 95% CI 1.40–11.95, p = 0.003) were independent predictive factors of a shorter OS (Table 3). For DFS, serous histological subtype (serous vs. non-serous: HR 2.29, 95% CI 1.18–4.43, p = 0.014), higher FIGO stage (III/IV vs. I/II: HR 9.40, 95% CI 4.23–20.86, p < 0.0001), intravascular tumor thrombus (Yes vs. No: HR = 2.00, 95% CI 1.07–3.75, p = 0.030), elevated serum CA125 (≥ 35 U/mL vs. < 35 U/mL: HR 5.90, 95% CI 1.43–24.32, p= 0.014) and over-expression of APOBEC3B (+++/++ vs. ± : HR 5.18, 95% CI 2.48–10.80, p< 0.0001) were significantly associated with worse DFS in the univariate analyses (Table 3). In multivariate analyses, higher FIGO stage (III/IV vs. I/II: HR 9.75, 95% CI 2.89–32.90, p< 0.0001) and higher expression of APOBEC3B (+++/++ vs. ± : HR 4.23, 95% CI 1.60–11.17, p= 0.004) were independent predictive factors of worse DFS (Table 3).

**Table 3:** Univariate and multivariate analyses of factors associated with overall survival

Variables	Overall survival		Disease-free survival	
	HR (95% CI)	p value	HR (95% CI)	p value
Univariate analyses				
Age (> 50 vs. ≤ 50)	1.10 (0.63–1.91)	0.734	1.18 (0.70–2.00)	0.537
Histological subtype (serous vs. non-serous)	2.24 (1.12–4.47)	*0.023*	2.29 (1.18–4.43)	*0.014*
FIGO stage (III/IV vs. I/II)	7.89 (3.53–17.60)	*< 0.0001*	9.40 (4.23–20.86)	*<0.0001*
Histological grade (G2/G3 vs. G1)	1.36 (0.72, 2.54)	0.341	1.44 (0.79–2.63)	0.239
Intravascular tumor thrombus (Yes vs. No)	2.27 (1.20–4.29)	*0.012*	2.00 (1.07–3.75)	*0.030*
CA 125 (≥ 35 U/mL vs. < 35 U/mL)	5.45 (1.30–22.56)	*0.019*	5.90 (1.40–24.32)	*0.014*
APOBEC3B (+++/++ vs. ±)	6.34 (2.80–14.38)	*< 0.0001*	5.18 (2.40–10.80)	*< 0.0001*
Multivariate analyses				
FIGO stage (III/IV vs. I/II)	7.89 (2.33–26.70)	*0.001*	9.75 (2.89–32.90)	*< 0.0001*
APOBEC3B (+++/++ vs. ±)	5.18 (1.40–11.95)	*0.003*	4.23 (1.60–11.17)	*0.004*

*CA 125* cancer antigen 125, *FIGO* Federation of Gynecologists and Obstetricians, *HR* hazard ratio, *95% CI* 95% confidence interval

### Effects of APOBEC3B expression on the viability of ovarian tumor cells

**Fig. 3:**
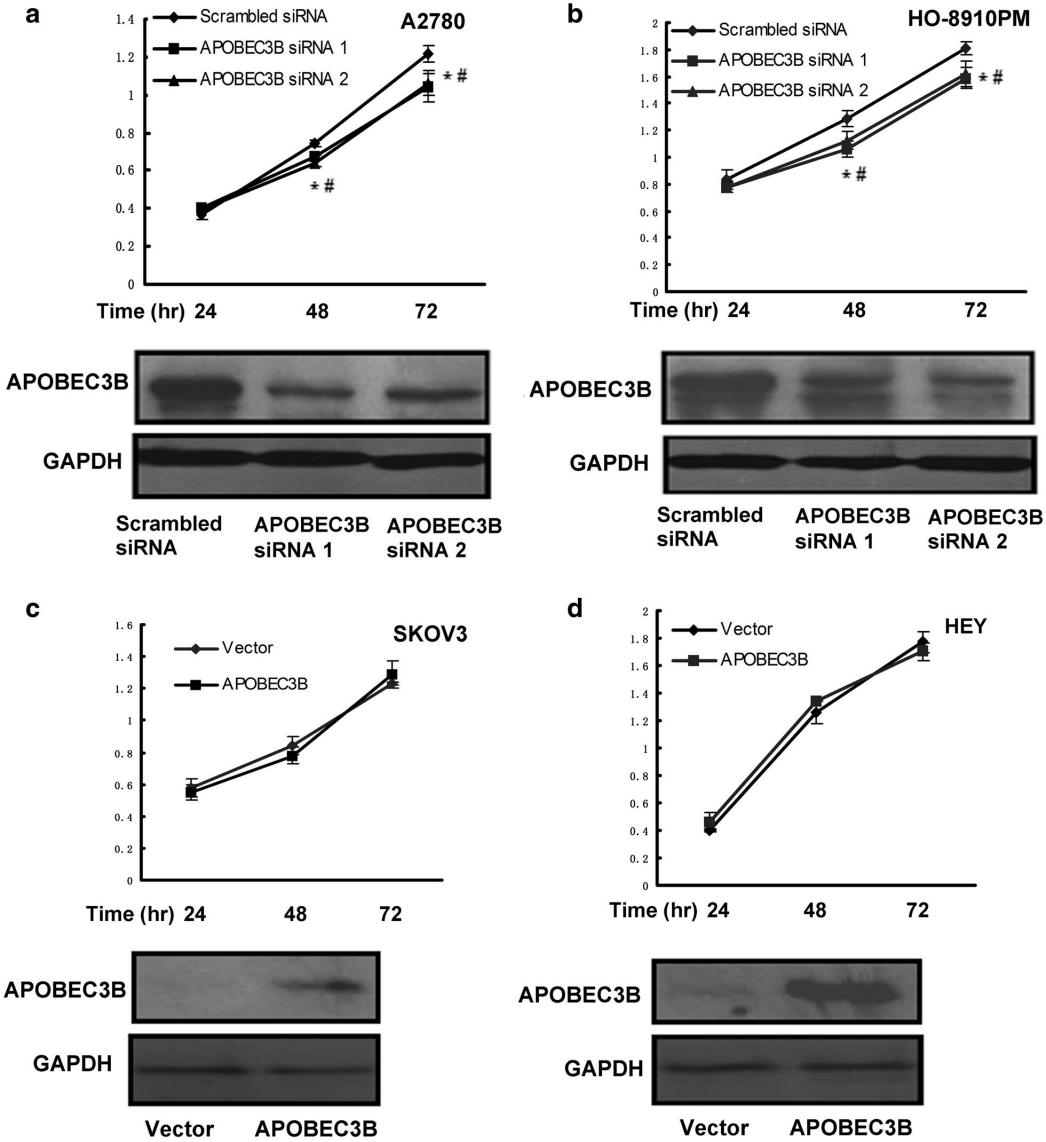
The effects of APOBEC3B on cell viability at different time point (24 hr, 48 hr, and 72 hr) of ovarian cancer cell lines. **a** and **b** A2780 and HO-8910PM cells were transfected with scrambled siRNA, APOBEC3B siRNA 1 or APOBEC3B siRNA 2, cell viability and APOBEC3B expression were detected by SRB assay (the upper panels) and western blotting analysis (the lower panels), respectively. **c** and **d** SKOV3 and HEY cells were transfected with Vector or pLenti-APOBEC3B, cell viability and APOBEC3B expression were detected by SRB assay (the upper panels) and western blotting analysis (the lower panels), respectively. Data are presented as mean ± SEM (n = 3) *p < 0.05, compared with control cells. A representative blot of triplicate blots is shown. GAPDH was used as the loading control

As APOBEC3B has been indicated in cancer progression [10], we then observed whether APOBEC3B promoted cell viability of human ovarian cancer cells. Four ovarian cancer cell lines with different endogenous expression of APOBEC3B were chosen. It has been shown that the expression of APOBEC3B in SKOV3 and HEY cells was lower, while the expression of APOBEC3B in A2780 and HO-8910PM was higher [15]. As shown in Fig. 3a, b, APOBEC3B siRNA caused a dramatic decrease in *APOBEC3B* expression in human A2780 and HO-8910PM ovarian cancer cells and knocking-down of APOBEC3B resulted in a modest decrease in cell viability. However, pLenti-APOBEC3B transfection could not improve cell viability of SKOV3 and HEY cells (Fig. 3c, d). Presumably, APOBEC3B over-expression could not improve cell viability directly but it may be crucial for the maintenance of cell viability.

## Discussion

Our study results showed that *APOBEC3B* expression was one of the independent prognostic factors to predict OS and DFS of ovarian cancer patients. In addition, the in vitro data from our study showed that knockdown of *APOBEC3B* expression affected ovarian cancer cell viability. Taken together, our study indicated that *APOBEC3B* expression may play an important role in viability and survival of ovarian cancer.

Ovarian cancer is a heterogeneous disease with a high degree of genomic instability, pro-/antitumor immunity and inflammation, and remains to be the most lethal gynecologic cancer [19]. Survival outcomes are also far from satisfactory, about 70% of patients diagnosed with advanced-stage ovarian cancer will suffer from recurrence due to high chemotherapy resistance or therapeutic failure [20]. It is of immense importance to identify biomarkers that can predict the prognosis of ovarian cancer patients, as well as help direct the use of tailored therapies.

APOBEC mutation pattern was identified in several cancer types including breast, bladder, cervical, head and neck and lung cancers. APOBEC family members can target host genomic DNA, generating signature mutation clusters in the genome. It is reported that the APOBEC-mediated mutagenesis was correlated with APOBEC mRNA levels, particularly with APOBEC3B [10, 11]. APOBEC3B is hypothesized to be the principal cancer driver of A3-catalyzed somatic mutations, and plays a prominent role in the genesis and evolution of various cancers. As part of the innate immune system, which plays a key role in combating exogenous infection especially viral infection, APOBEC3B expression is stimulated by a complex network of innate immune responses involving components such as interferons, interleukins, and Toll-like receptors [7]. APOBEC3B can cause C-to-T transition in carcinogenesis [21].

Studies have shown that *APOBEC3B* expression is elevated in a variety of cancer tissues and cell lines, compared to the low levels in the corresponding normal human tissues [10, 21, 22]. In the genome of cervical cancer patients, APOBEC3B protein over-expression was correlated with the enrichment of *APOBEC3B* mutation signature [11]. What’s more, in vitro study showed estrogen can directly activate *AID* mRNA expression in an ovarian cancer cell line [23]. As for ovarian cancer, study has shown that high *APOBEC3B* expression correlates with C-to-A and C-to-G transversion mutations within 5′-TC dinucleotide motifs in the genomes of early-stage high-grade serous ovarian cancer, indicating APOBEC3B may play a role in serous ovarian cancer genomic instability [15]. Studies have reported that higher levels of *APOBEC3B* expression is associated with decreased survival rates among estrogen receptor-positive (ER+) breast cancer patients [12, 24]. Furthermore, elevated *APOBEC3B* expression is correlated with a declined efficacy of tamoxifen (TAM) therapy in recurrent ER + breast cancer patients [25]. In consistent with previous studies, our multivariate results showed that higher expression of APOBEC3B was independently associated with worse survival of ovarian cancer patients. Because of its tumorigenic role in promoting mutational processes, *APOBEC3B* has clinical potential to serve as novel therapeutic target. It is likely that chemical probes and therapeutic molecules targeting *APOBEC3B*, when used in combination with existing cancer therapies, will slow or possibly prevent metastasis, development of drug resistance, and therapy failure in human cancers [26]. It has been well established that APOBEC3B is a molecular driver of mutagenesis in various human cancers [22]. Its aberrant expression leads to unexpected clusters of mutations in the majority of cancers [22]. In our present study, we observed that APOBEC3B knockdown affected cell viability, which may attribute to genomic instability caused by aberrant expression of APOBEC3B. However, we did not observe APOBEC3B over-expression in SKOV3 and HEY cells improved cell viability. Notably, the cell lines in our study were transiently transfected with pLenti-APOBEC3B construct. Presumably, stably transfected cells with APOBEC3B over-expression may demonstrate gene mutations and more dramatic cellular biological behaviors.

In the present study, we investigated the tumorigenic role of APOBEC3B in ovarian cancer. In fact, the ovarian cancer cells may express several AID/APOBEC family members, which may also contribute to the tumorigenesis of ovarian cancer. Further investigation is in progress to detect whether and how the other members of AID/APOBEC family are involved in the tumorigenesis of ovarian cancer. In addition, due to the high degree of heterogeneity in ovarian cancer, AID/APOBEC family members may act in a patient-specific manner, alone or in combination with tumor-infiltrating immune cells. Moreover, we observed a statistically significant association of APOBEC3B expression with FIGO stage, but did not detect any association between APOBEC3B expression and histological subtype nor histological grade. It could possibly due to a lack of statistical power resulted from limited sample size (n = 88, especially there were 22 missing values for histological grade). Therefore, future studies with adequate sample size are needed to understand the role of APOBEC3B in ovarian cancer progression.

## Conclusions

In conclusion, our study showed that over-expression of APOBEC3B is associated with the development and prognosis of ovarian cancer patients, possibly through influencing viability of ovarian cancer. Therefore, APOBEC3B is a potential therapeutic target which may contribute to the development of new ovarian cancer treatment strategies.

## Abbreviations

APOBEC3B: apolipoprotein B mRNA editing enzyme catalytic subunit 3B
ATCC: American Type Culture Collection
CA 125: cancer antigen 125
DFS: disease-free survival
ER+: estrogen receptor-positive
FIGO: Federation of Gynecologists and Obstetricians
HE: hematoxylin and eosin
HR: hazard ratios
HRP: horseradish peroxidase
OS: overall survival
SD: standard deviation
SRB: Sulforhodamine B
TAM: tamoxifen
TMA: tissue microarray
95% CI: 95% confidence interval
